# The complete chloroplast genome of *Primula vialii* (Primulaceae), an ornamental plant

**DOI:** 10.1080/23802359.2023.2202268

**Published:** 2023-05-31

**Authors:** Yunqi Liu, Li Zhang, Shubao Wang, Rui Li, Yuan Huang

**Affiliations:** School of Life Sciences, Yunnan Normal University, Kunming, P. R. China

**Keywords:** Chloroplast genome, *Primula vialii*, phylogenomic analysis

## Abstract

*Primula vialii* Delavay ex Franch. (1905) is an alpine species with an ornamental value. In this study, we sequenced, assembled, and annotated the chloroplast genome of *P. vialii.* The results showed that it was a double-stranded, closed circular DNA with 154,897 bp in length, comprising a small single-copy (SSC) region of 17,766 bp, a large single-copy (LSC) region of 85,379 bp and a pair of inverted repeat (IR) regions of 25,876 bp. A total of 113 unique genes were annotated, including 79 protein-coding genes, 30 tRNA genes, and 4 rRNA genes. The phylogenetic analysis revealed that *P. vialii* is closely related to *Primula flaccida.* The cp genomic data will be useful for systematics and evolutionary studies of *Primula*.

## Introduction

**Figure 1.**
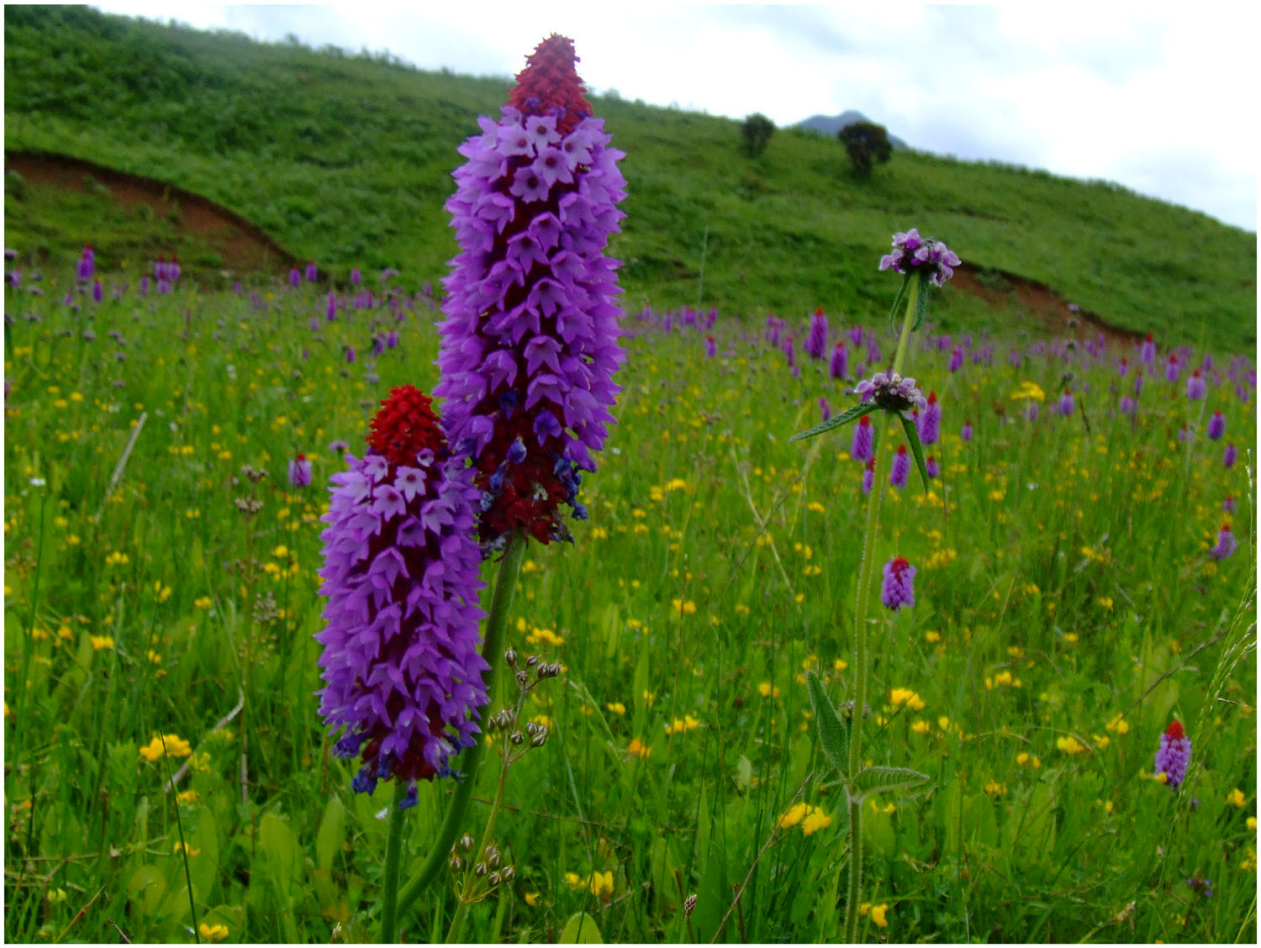
Species reference image of the *Primula vialii.* This image is provided by the corresponding author Yuan Huang.

*Primula vialii* Delavay ex Franch. (1905) is a perennial herb in the genus *Primula* (Primulaceae), which widely distributed in wet meadows and valleys at an altitude of 2800–4000 m in Northwest Yunnan and Southwest Sichuan of China (Hu and Kelso 1996). With its blue and red spires of flowers (Figure 1), this extraordinary species presents an unique feature in *Primula* (Richards 2003). Because of its dark green basal leaves, it is invariably used as an ornamental foliage plant during vegetative growth period. However, previous studies have focused on tissue culture and rapid propagation (Li et al. 2005) of *P. vialii* rather than systematics and evolutionary studies. Here, we have sequenced and analyzed the chloroplast (cp hereafter) genome of *P. vialii*, which contributes to provide more cp genome genetic information further evolutionary research and phylogenetic study of *Primula*.

## Materials and methods

Fresh leaves of an individual *P. vialii* were collected from the Alpine Botanical Garden of Lijiang City, Yunnan Province, China (26.997°N, 100.201°E). The voucher specimen (accession No. HY-12) was deposited at the Herbarium of Yunnan Normal University (Kunming, China; Jianlin Hang, hjlyuun@163.com). Total genomic DNA was extracted from the fresh leaves using the modified CTAB method (Porebski et al. 1997) and stored in Huang’s Lab of Yunnan Normal University. The total DNA was fragmented into 300 bp short sequences following the manufacture’s protocoal (Illumina Inc., USA) to construct a DNA library, then sequenced on the Illumina Hiseq X Ten sequencing platform. We obtained 18,438,252 filtered reads of paired-end sequences (2 × 150 bp) and assembled the complete cp genome using NOVOPlasty v4.3.1 (Dierckxsens et al. 2017). To verify the accuracy of the assembly, we further mapped our clean reads back to the assembled cp genome to assess the depth of coverage (Figure S1). Taking the cp genome of *Primula flaccida* (Genbank accession No. NC_053595) as the reference sequence, the assembled genome was annotated using Geneious v2020.1.1 software (Kearse et al. 2012). The annotated cp genome sequences of *P. vialii* were deposited in the GenBank database under accession No. ON584545. The genome map and cis/trans-splicing genes map (Figure S2) was drawn using the CPGView program (http://www.1kmpg.cn/cpgview).

In order to investigate the phylogenetic relationship of *P. vialii* in the genus *Primula*, we downloaded 39 *Primula* cp genomes and 5 Primulaceae cp genomes (*Glaux maritima* (Liu et al. 2021), two *Lysimachia* species and two *Androsace* species) as outgroups from NCBI. The 45 cp genome sequences were aligned by software MAFFT v7.47 (Katoh and Standley 2013), then constructed a maximum likelihood tree using the software IQ-TREE 2 (Katoh and Standley 2013; Minh et al. 2020) under TVM + F + R3 best-fit model according to Bayesian information criterion (Kalyaanamoorthy et al. 2017). Branch supports were tested using ultrafast bootstrap (UFBoot) (Hoanget al. 2018) and SH-like approximate likelihood ratio test (SH-aLRT) (Guindon et al. 2010) with 10,000 replicates.

## Results

**Figure 2.**
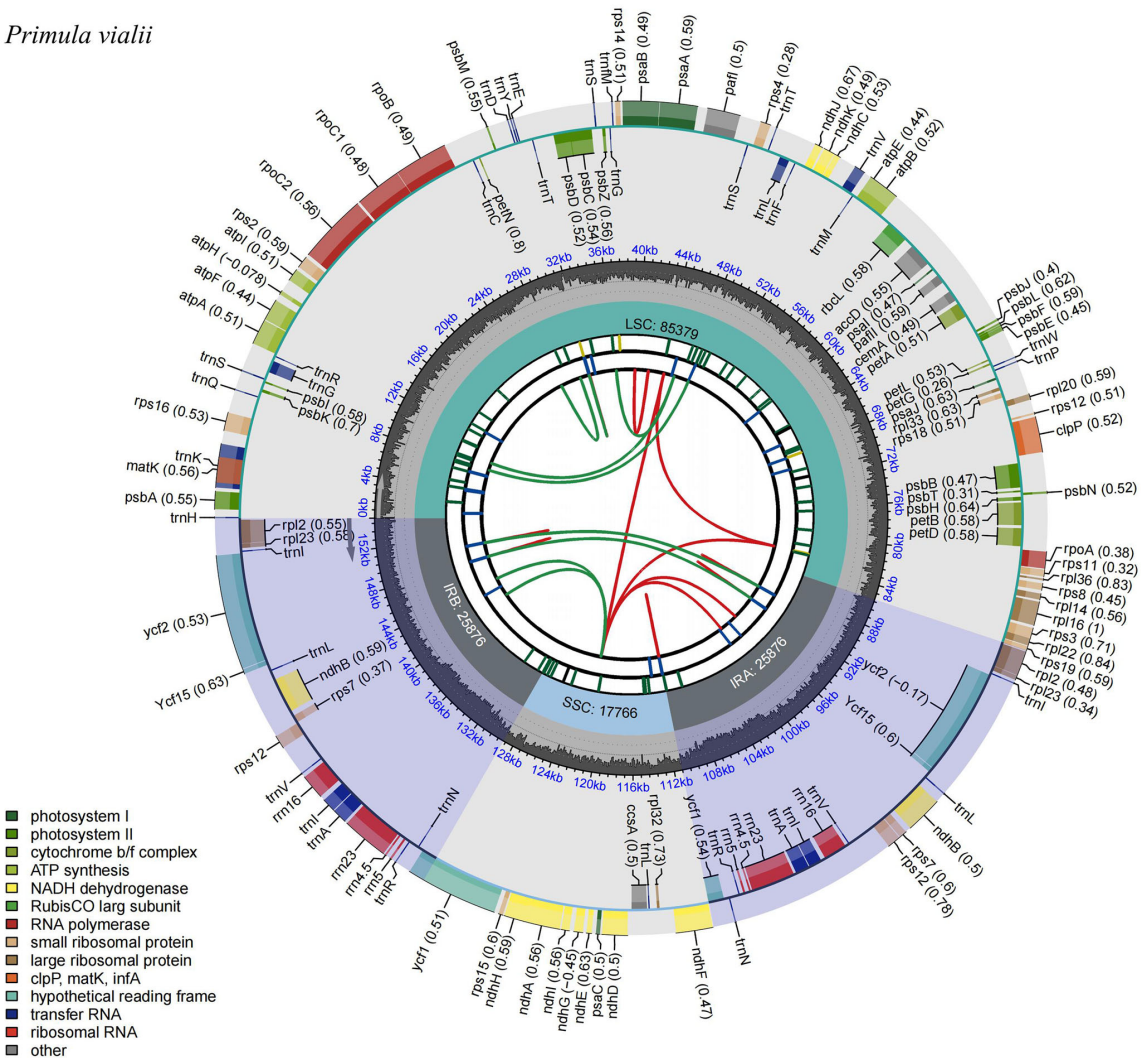
Genomic map of *Primula vialii* chloroplast genome generated by CPGview (http://www.1kmpg.cn/cpgview/). The species name is shown in the left top corner. The map contains six tracks in default. From the center outward, the first track shows the dispersed repeats. The dispersed repeats consist of direct and Palindromic repeats, connected with red and green arcs. The second track shows the long tandem repeats as short blue bars. The third track shows the short tandem repeats or microsatellite sequences as short bars with different colors. The small single-copy (SSC), inverted repeat (IRA and IRB), and large single-copy (LSC) regions are shown on the fourth track. The GC content along the genome is plotted on the fifth track. The base frequency at each site along the genome will be shown between the fourth and fifth tracks. The genes are shown on the sixth track. The optional codon usage bias is displayed in the parenthesis after the gene name. Genes belonging to different functional groups are color-coded. Genes drawn inside the circle are transcribed clockwise, and those outside are transcribed counterclockwise.

**Figure 3.**
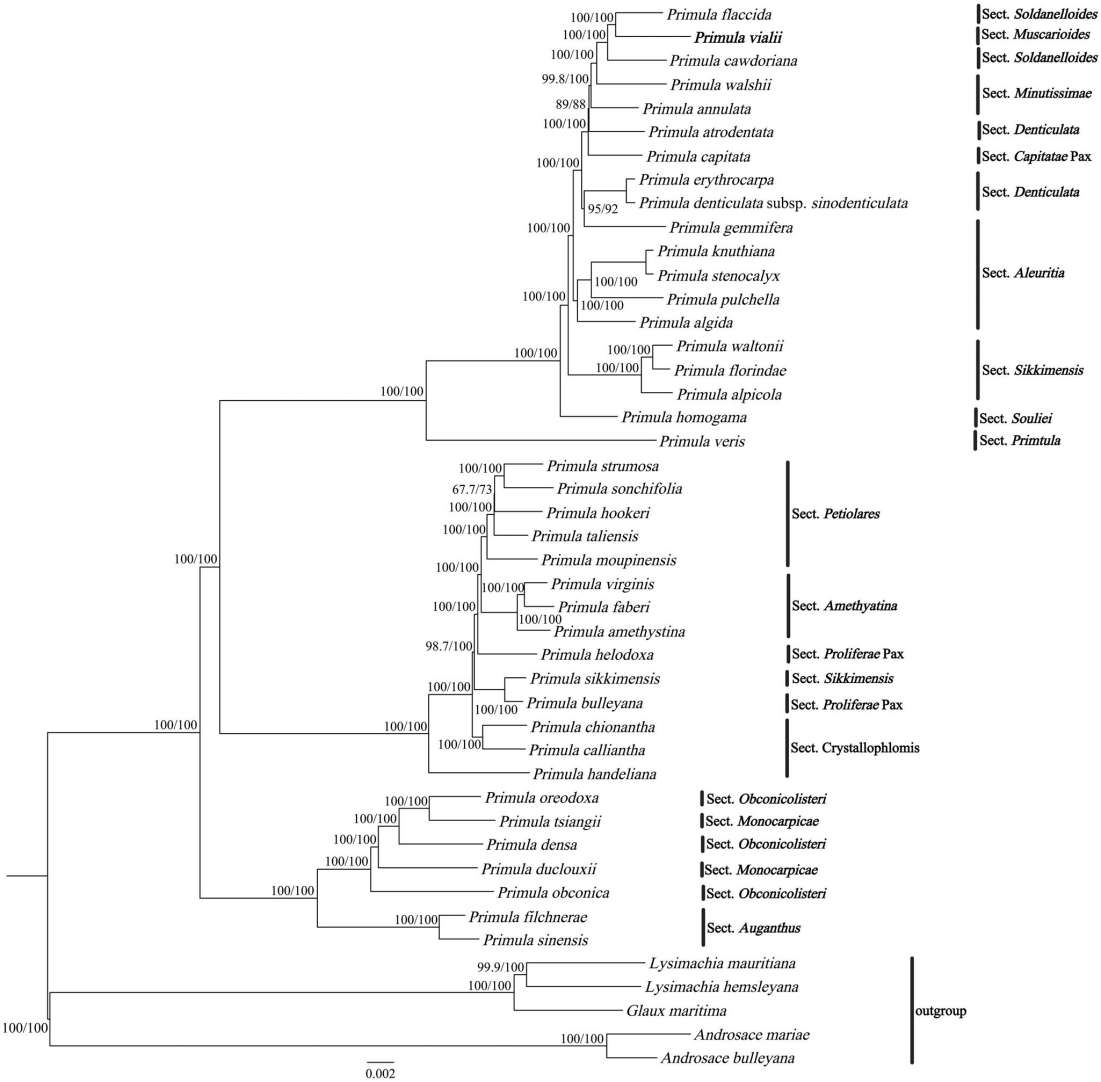
ML phylogenetic tree of *Primula vialii* and 44 Primulaceae species based on complete chloroplast genomes, the branch supports values were reported as SH-aLRT/UFBoot. The bolded font represents the chloroplast genome of *P. vialii* in this study. The following sequences were used: *Primula flaccida* NC_053595 (Wang et al. 2021), *Primula cawdoriana* NC_053586 (Wang et al. 2021), *Primula walshii* NC_053597 (Wang et al. 2021), *Primula annulata* NC_053608 (Wang et al. 2021), *Primula atrodentata* NC_053587 (Wang et al. 2021), *Primula capitata* NC_053589 (Wang et al. 2021), *Primula erythrocarpa* NC_053598 (Wang et al. 2021), *Primula denticulata* subsp*. sinodenticulata* NC_050247, *Primula gemmifera* NC_053590 (Wang et al. 2021), *Primula knuthiana* NC_039350 (Ren et al. 2018); *Primula stenocalyx* NC_058249 (Guo et al. 2021b), *Primula pulchella* NC_050246, *Primula algida* NC_053582 (Wang et al. 2021), *Primula waltonii* NC_058808, *Primula florindae* NC_053579 (Wang et al. 2021), *Primula alpicola* NC_053588 (Wang et al. 2021), *Primula homogama* NC_054305 (Sun et al. 2021), *Primula veris* NC_031428, *Primula strumosa* NC_053599 (Wang et al. 2021), *Primula sonchifolia* NC_053594 (Wang et al. 2021), *Primula hookeri* NC_053593 (Wang et al. 2021), *Primula taliensis* NC_053601 (Wang et al. 2021), *Primula moupinensis* NC_050244, *Primula virginis* NC_053581 (Wang et al. 2021), *Primula faberi* NC_053576 (Wang et al. 2021), *Primula amethystina* NC_053577 (Wang et al. 2021), *Primula helodoxa* NC_046771 (Zhang, Chen, et al. 2019), *Primula sikkimensis* NC_050243, *Primula bulleyana* NC_046947 (Chen, Zhang, et al. 2019), *Primula chionantha* NC_053583, (Wang et al. 2021), *Primula calliantha* MZ054238 (Yang et al. 2021), *Primula handeliana* NC_039348 (Ren et al. 2018), *Primula oreodoxa* NC_050848, *Primula tsiangii* NC_046755 (Chen, Yan, et al. 2019), *Primula densa* NC_058262 (Zhong et al. 2019), *Primula duclouxii* NC_058263 (Zhong et al. 2019), *Primula obconica* NC_046415 (Zhang, Yuan, et al. 2019), *Primula filchnerae* NC_051972 (Lu et al. 2020), *Primula sinensis* NC_030609 (Liu et al. 2016), *Lysimachia mauritiana* NC_060700 (Lee et al. 2022), *Lysimachia hemsleyana* NC_052863 (Ying et al. 2019), *Glaux maritima* NC_059901 (Liu et al. 2021), *Androsace mariae* NC_051991 (Guo et al. 2021a), *Androsace bulleyana* (NC_034641).

The cp genome of *P. vialii* was assembled as a double-stranded, closed-circular DNA molecule of 154,897 bp in length (Figure 2), with an average coverage of 864.9 and GC contents of 36.9%. It has a typical quadripartite structure and comprises a large single-copy (LSC) region of 85,379 bp and a small single-copy (SSC) region of 17,766 bp, separated by two inverted repeat (IR) regions of 25,876 bp each. The GC contents of these four regions are 34.8%, 30.3%, 42.6%, 42.6%, respectively. In total, the cp genome has 132 genes, including 87 protein-coding genes, 37 tRNA genes, and 8 rRNA genes, of which 113 genes, 79 protein-coding genes, 30 tRNA genes, 4 rRNA genes are unique, respectively. Most of these genes are single-copy genes, while 8 protein-coding genes, 7 tRNA genes, and 4 rRNA genes were duplicated in the IR regions. The phylogentic tree (Figure 3) indicated the relationship between *Primula, Androsace*, *Lysimachia,* and *Glaux* in Primulaceae, and 40 *Primula* species formed a monophyletic clade. In addition, *P. vialii* is closely related to *P. flaccida* and *P. cawdoriana* in Sect. *Soldanelloides.*

## Discussion and conclusion

Within *Primula* clade, *P. vialii*, *P. flaccida,* and *P. cawdoriana* formed a monophyletic clade and were sister to each other (Figure 3), which is consistent to previous studies based on noncoding cp DNA sequence (Mast et al. 2001). Mast (2001) concluded that *P. vialii* belongs to Sect. *Muscarioides*, and members of Sect. *Muscarioides* are interdigitated with members of Sect. *Soldanelloides*. Although we reported the first complete cp genome of Sect. *Muscarioides* in this study, other complete cp genomes of Sect. *Muscarioides* are not sequenced yet. Therefore more phylogenetic researches are needed to exactly confirm the relationship of Sect. *Muscarioides* and Sect. *Soldanelloides*. Overall, the complete cp genome of *P. vialii* can be subsequently used for phylogenetic, taxonomic, and evolutionary studies of Sect. *Muscarioides.*

## Supplementary Material

Supplemental Material

Supplemental Material

## Data Availability

The genome sequence data that support the findings of this study are openly available in GenBank of NCBI at [https://www.ncbi.nlm.nih.gov/] under accession no. ON584545. The associated BioProject, SRA, and Bio-Sample numbers are PRJNA841839, SRR19391427, and SAMN28626130, respectively.
