# Supplementary figures and images for: The complete chloroplast genome of Primula vialii (Primulaceae), an ornamental plant
Source: Mitochondrial DNA B Resour. 2023 May 31;8(5):619–23. doi: 10.1080/23802359.2023.2202268 (PMC10236967; doi:10.1080/23802359.2023.2202268)

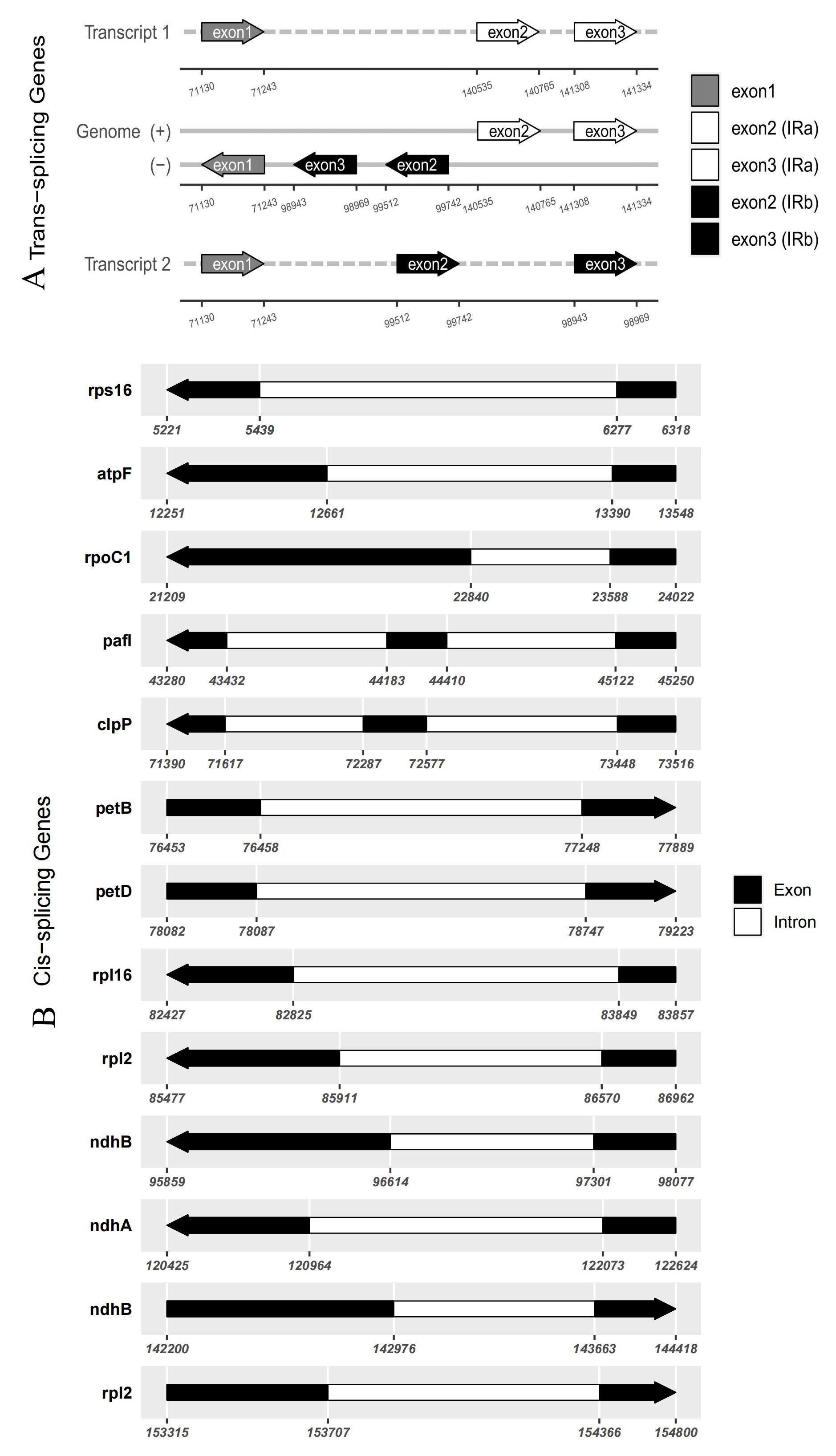

Supplement: Supplemental Material [file TMDN_A_2202268_SM5717.jpg]

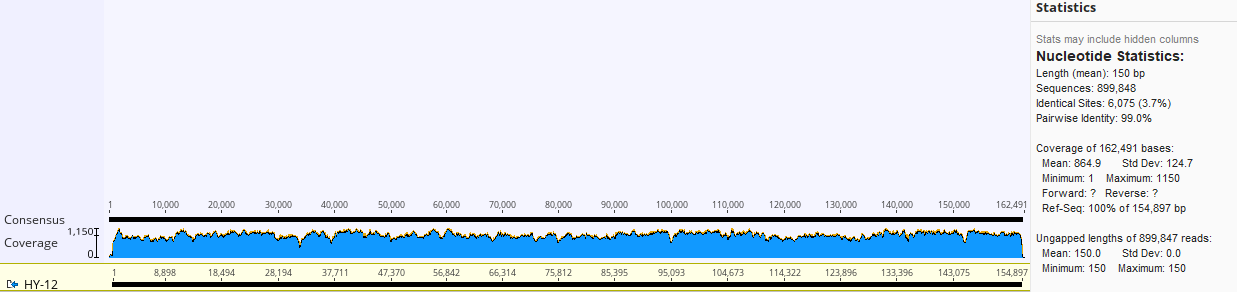

Supplement: Supplemental Material [file TMDN_A_2202268_SM5711.png]
